# Noninvasive Assessments of Mitochondrial Capacity in People with Mitochondrial Myopathies

**DOI:** 10.3390/muscles3040033

**Published:** 2024-11-26

**Authors:** Kevin K. McCully, Hannah M. Bossie, Fran D. Kendall

**Affiliations:** 1 Department of Kinesiology, University of Georgia, Athens, GA 30605, USA; 2 Intuitive Surgical, Sunnyvale, CA 30086, USA; hannah.bossie@intusurg.com; 3 Virtual Medical Practice, Atlanta, GA 30338, USA; fdk@vmpgenetics.com

**Keywords:** near-infrared spectroscopy, NIRS, Kearns–Sayre syndrome, MELAS, skeletal muscle

## Abstract

People affected by mitochondrial myopathies (MITOs) are thought to have impaired skeletal muscle oxygenation. The aims of this study were to measure skeletal muscle mitochondrial capacity in MITO participants and able-bodied (AB) participants and evaluate the influence of muscle-specific endurance training in one MITO participant. Participants (n = 7) with mitochondrial disease and controls (n = 9) were tested (ages 18–54 years). Mitochondrial capacity (mVO_2_max) was measured using the rate constant of recovery of oxygen consumption (mVO_2_) after exercise in the forearm flexor muscles with near-infrared spectroscopy (NIRS). One MITO participant was tested before and after performing 18 forearm exercise sessions in 30 days. There were no differences between MITO and AB participants in mVO_2_max (MITO: 1.4 ± 0.1 min^−1^; AB: 1.5 ± 0.3 min^−1^; *p* = 0.29), resting mVO_2_ (MITO: −0.4 ± 0.2%/min; AB: −0.3 ± 0.1%/min; *p* = 0.23), or initial post exercise oxygen consumption rates (MITO: 4.3 ± 1.2%/min; AB: 4.4 ± 1.4%/min; *p* = 0.9). Exercise oxygen desaturation was greater in MITO (39.8 ± 9.7% range) than in AB (28 ± 8.8% range) participants, *p* = 0.02. The MITO participant who trained increased her mitochondrial capacity (58%) and muscle-specific endurance (24%) and had reduced symptoms of muscle fatigue. We found no evidence supporting in vivo impairment of forearm muscle mVO_2_max in genetically confirmed MITO participants. This is consistent with studies that report increased mitochondrial content, which offsets the decrease in mitochondrial function. Positive muscle adaptations to endurance training appear to be possible in people with MITOs. Characterization of study populations will be important when interpreting the relationship between in vivo mitochondrial capacity and mitochondrial disease.

## 1. Introduction

Mitochondrial diseases (MITOs) are among the most prevalent inheritable diseases in which the mitochondria within the body’s cells have an impaired ability to produce usable energy in the form of ATP [1,2,3]. Mitochondrial diseases typically result from a mutation or deletion in nuclear or mitochondrial DNA and typically impact organ systems with high energy requirements, most commonly including the brain and nervous system, heart, kidneys, liver, eyes, ears, and skeletal muscles [4]. The symptoms of mitochondrial myopathies can be highly variable, as well as comprehensive. Symptoms may include ophthalmoplegia, blindness, neuropathy, ataxia, stroke-like episodes, cardiomyopathy/arrhythmias, blood dyscrasias, hearing loss, muscle weakness, exercise intolerance, low muscle tone, migraines, seizures, chronic fatigue, kidney and liver disease, developmental issues such as autism, diabetes, and other endocrine issues [5]. MITOs can be genetically inherited or result from a spontaneous mutation.

The diagnostic process for mitochondrial myopathies is extensive and typically includes a battery of tests depending on what clinical symptoms are presented [4]. This process can include tests for biochemical abnormalities in both blood and urine, CSF, genetic analysis, exercise testing, radiographic studies and comprehensive history [5]. Diagnostic testing also commonly includes a skeletal muscle biopsy, which is an invasive although routine procedure. Post exercise measurements of blood lactate and high-energy phosphates using magnetic resonance spectroscopy (MRS) have been used, although these measures are not considered reliable diagnostic tools [6,7]. MRS measurements of brain lactate can also be used diagnostically but do not provide information on muscle status. Due to the invasiveness and sometimes inconclusiveness of the diagnostic process, it could be helpful to provide evidence of mitochondrial dysfunction.

Near-infrared spectroscopy (NIRS) is a noninvasive technology that measures the relative concentrations of oxygenated and deoxygenated myoglobin and hemoglobin within skeletal muscle, based on their differential absorbencies of infrared light [8,9,10]. NIRS measures have previously demonstrated that people affected by MITOs have impaired skeletal muscle oxygen consumption, based on tissue oxygenation before, during, and after exercise, and this has even been proposed as a diagnostic tool for this population [11,12,13]. While it is generally hypothesized that mitochondrial defects will impair mitochondrial function, it has also been suggested that mitochondrial biogenesis could compensate so that skeletal muscle mitochondrial capacity (a combination of function and number) could remain unimpaired [14]. Exercise training has generally been shown to induce positive adaptations in people with MITOs [15,16,17]. However, most of the changes have been seen in whole-body exercise capacity and not specifically linked to the training of specific muscles.

The purpose of this study was to assess skeletal muscle mitochondrial capacity in people with genetically confirmed skeletal muscle mitochondrial myopathies and compare them to controls. We used an NIRS-based measurement of the recovery of oxidative metabolism after a short bout of exercise to characterize skeletal muscle mitochondrial capacity [18,19,20]. In addition, one participant with a MITO performed one month of muscle-specific endurance training centered on the forearm. We hypothesized that MITO participants would have impaired skeletal muscle oxidative capacity reflected in lower recovery rate constants, hyperoxygenation during exercise, and lower resting metabolic rates. Furthermore, we hypothesized that endurance training would increase muscle mitochondrial capacity in a patient with a MITO.

## 2. Results

MITO participants varied, with different genetic mutations and clinical syndromes including mitochondrial myopathy (MM), mitochondrial encephalomyopathy, lactic acidosis, and stroke-like episodes (MELAS), and Kearns–Sayre Syndrome (KSS). There were no statistically significant differences between MITO and AB participants’ ATT, height, weight, age, BMI, or physical activity level as assessed by the IPAQ questionnaire (Table 1).

**Figure 1:**
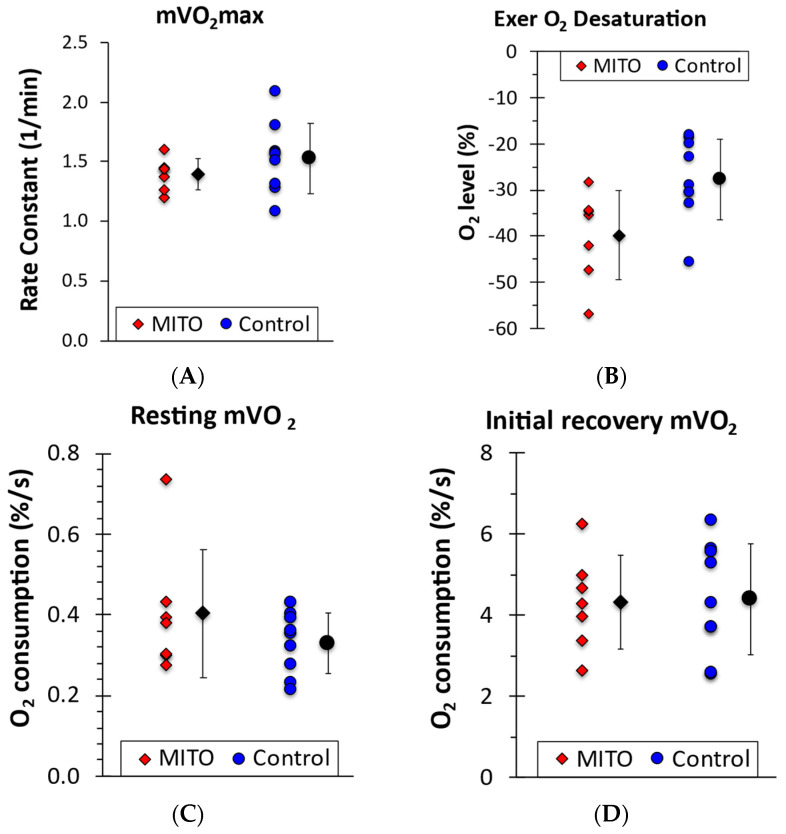
(**A**) Mitochondrial capacity, (**B**) maximum exercise O_2_ desaturation, (**C**) resting metabolism, and (**D**) post exercise metabolic rate. Individual values in red and blue, with black symbols indicating means and SD.

Figure 1 shows the results of the NIRS testing. There were no differences between MITO and AB participants in mVO_2_max (*p* = 0.31). In addition, there were no differences in resting muscle oxygen consumption (*p* = 0.35), or initial post exercise mVO_2_ (*p* = 0.89). Both groups showed muscle oxygen desaturation during the exercise, which was greater in the MITO group than the control group (*p* = 0.04).

**Figure 2:**
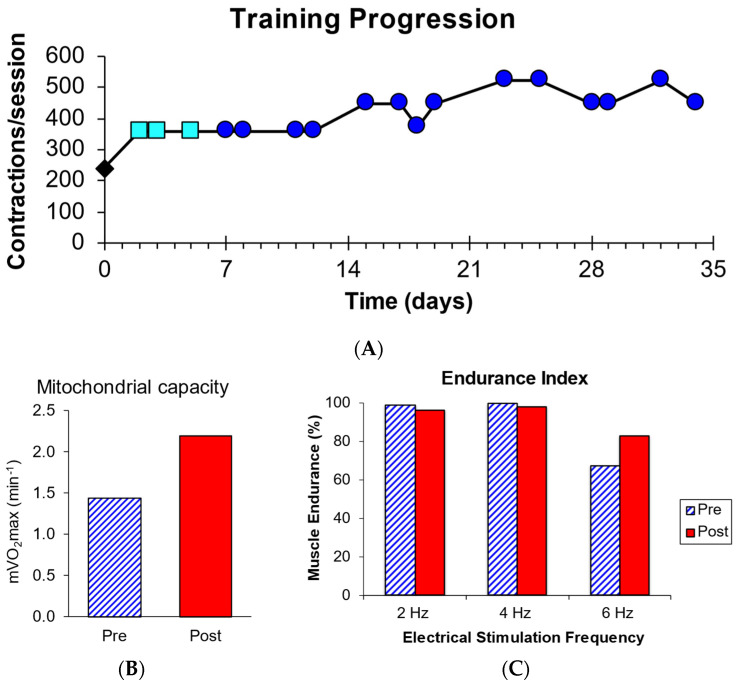
(**A**) The number of contractions per session over the one-month training period. The initial training session (black diamond used 2.28 kg, the next sessions (light blue squares) used 1.38 kg, and the remaining sessions (dark blue circles) used 1.82 kg. (**B**) mVO_2_max increased after training. (**C**) muscle endurance index increased at 6 Hz stimulation after training.

One participant from the MITO group performed forearm exercise training, which started with 20 min of forearm exercise consisting of 240 contractions with a 2.28 kg weight. This weight was deemed to be too heavy by the participant, and the next three sessions used 1.38 kg. The participant then increased the weight lifted to 1.82 kg for the remaining exercise sessions and finished performing 450 contractions per session (Figure 2A). Muscle mitochondrial capacity increased by 58% (Figure 2B), and muscle-specific endurance increased by 24% following training (Figure 2C). Grip strength was 19 kg prior to training and 21 kg at the end of training. The subject’s description of the training sessions was recorded, and selected responses are shown in Table 2. The participant’s sensations of muscle fatigue decreased dramatically over the course of training.

## 3. Discussion

We did not find evidence of impaired muscle metabolism in participants with genetically documented mitochondrial defects, as measured by NIRS methodology. This conclusion was based on the lack of differences between MITO and control groups in mVO_2_max, which is the rate of recovery of muscle metabolism after exercise. In addition, the participants with MITOs had resting muscle metabolism values that were not different from the control group. With exercise, participants in the MITO group showed muscle desaturation that was not less than that of the control group. The MITO group had similar initial post exercise rates of muscle metabolism as the control group. All of these measurements indicate that the muscles from the MITO participants were consuming comparable amounts of oxygen as the healthy control participants.

The results of this study were not consistent with several previous studies that used a variety of methodologies. Whole-body exercise studies have reported reduced oxygen consumption values and cardiac output relative to oxygen consumption ratios in MITO patients compared to controls [17]. Excessive production of lactate during exercise has been proposed as a diagnostic marker of impaired mitochondrial function, although not all studies have found this approach to be useful [6]. Previous studies that examined mitochondrial capacity by phosphorous nuclear magnetic resonance (^31^P-MRS) have shown that PCr, Pi, and ADP recovery halftimes were significantly slower in mitochondrial myopathies than in controls [21,22]. However, metabolic heterogeneity in the response of MITO patients to exercise has been reported with ^31^P-MRS measurements [23].

Previous NIRS studies have shown hyperoxygenation at the onset of exercise in patients with MITOs [11] and have even proposed this measure as a supplementary diagnostic tool for mitochondrial myopathies [12,24]. This hyperoxygenation has been attributed to the working muscles’ restricted ability to extract oxygen from the blood, resulting in hyperkinetic circulation during exercise, with excessive oxygen delivery in comparison to oxygen uptake [11]. However, various studies have yielded inconsistent results, with some participants hyperoxygenating, some deoxygenating normally, some deoxygenating significantly less than normal, or some even deoxygenating for the first 30 s of exercise and then oxygenating [12,25].

It is not clear why our participants responded differently from those from other studies. One possible explanation for our results is that there was mitochondrial compensation, where skeletal muscle cells upregulate mitochondrial biogenesis to offset the reduced mitochondrial function. Wredenberg et al. [14] created a mouse model of mitochondrial myopathy (MITO) by disrupting the gene for mitochondrial transcription factor A (Tfam), which leads to the development of many of the hallmarks of mitochondrial myopathies, including ragged red muscle fibers, the accumulation of abnormal mitochondria, and deteriorating respiratory chain function. This mouse model had increased mitochondrial mass within the skeletal muscle and normal ATP production. Although these results are surprising, their rationale was that the impaired respiratory chain function was compensated for by this increase in mitochondrial mass. Additionally, in vitro assessments of muscle fatigue showed that the MITO model had lesser force output but did not fatigue more rapidly than controls, indicating that ATP production was sufficient. This study concluded that the pathophysiology of mitochondrial myopathies may not be largely driven by reduced mitochondrial ATP production as previously thought [14]. These results support the concept that the functional effects of mitochondrial myopathies might not be what we would presume due to compensation mechanisms such as increased mitochondrial mass.

Muscle compensation, whereby other adaptations in skeletal muscle occur to increase oxidative metabolism to respond (or in response) to impaired mitochondrial function, was reported by Aydin et al. [26]. This study found that MITO mice had increased mitochondrial calcium and decreased sarcoplasmic reticulum calcium, which is a compensatory mechanism to increase ATP production. The long-term effects of this compensation can result in cellular damage and muscle weakness [26]. This proposed compensation further corroborates the idea that mitochondrial myopathy patients may be able to compensate for their impaired mitochondrial function in order to maintain ATP production, yielding normal skeletal muscle mitochondrial capacity.

Although mitochondrial compensation theory may help to explain the results of this study, it does not account for the difference between this study and previous studies. Discrepancies with historical data may be linked to severity of disease in the different study patient populations. It could also be due to differences in physical activity levels, as muscle mitochondria are activity dependent. It is possible that studying muscle from the forearm, which is generally inactive, results in fewer changes in oxidative capacity than locomotory muscles in the leg, which are more active in healthy participants than in MITO patients. The diagnosis and treatment of MITOs has evolved to allow for the identification of affected patients earlier in their course [4]. The introduction of physical therapy and activity, a standard component of current care, and other interventions may be leading to a healthier population in general [27]. The variability in sub-populations of mitochondrial diseases and activity levels also make direct comparison to earlier publications difficult.

The participant with a MITO who performed the wrist flexion training protocol improved her mitochondrial capacity. The improvement of 58% was similar to improvements seen in young healthy adults (64%) and healthy older adults (45%) with similar training programs. This improvement was associated with an increase in muscle endurance during the six-Hertz stimulation portion of the muscle endurance test. In addition, the participant demonstrated more than a doubling of the number of contractions per training session, and markedly improved her self-reported symptoms of muscle fatigue after training. Our training case study is consistent with previous whole-body training studies that showed that endurance training improved whole-body oxygen consumption and exercise capacity in patients with MITOs [15,16,17]. Our results extend previous findings to suggest that patients with MITOs can upregulate their muscle mitochondrial capacity. Our training results also support the conclusions that normal baseline mitochondrial capacity in the forearm could be a result of compensation and increasing mitochondrial content.

There are a few methodological limitations in this study. Because muscle biopsies in our MITO subjects were taken from the thigh muscles, we had to assume that the mitochondrial defects in the muscle we tested (forearm) was consistent with the muscle that was used for the biopsy (vastus lateralis). We chose the forearm because it is easier to evaluate with the NIRS method, and it is an activity-independent muscle in that it is not involved with walking. Our study population primarily included women. Five of the seven MITO subjects were women, and eight of the nine able-bodied controls were women. Our previous studies have not reported sex differences in mVO_2_max, but it is possible that MITOs might influence women differently than men. Our study had an age range of 18 to 59 years, which could potentially influence the mVO_2_max measurements. The age range was similar between groups, and thus we do not feel that age was a significant variable in our study. Our training study only included one person, so it is possible that other people with MITOs would respond differently. Because our subjects were located more than 50 miles from our testing site, it was only feasible for one subject to perform the training protocol. Another limitation is that our clinical data for our MITO subjects were limited to standard clinical assessments, and additional research-related measurements would have helped to further characterize our subjects.

## 4. Materials and Methods

Participants with a genetically confirmed mitochondrial myopathy (MITO) were recruited for participation in this study (Table 3). To be eligible for the study, participants with a MITO had to have a genetically identified mitochondrial myopathy and be between the ages of 7 and 60 years. The exclusion criteria were as follows: mental impairment such that informed consent could not be obtained or that the subject would not be safe with the protocol, evidence of drug dependency or heavy alcohol use that would interfere with testing, evidence of any unstable medical condition that would make participation unsafe, the presence of significant vascular disease, any prior injury to the arm that would make voluntary exercise unsafe, and any motor impairment such that the participant would be unable to complete 5–20 s of forearm curls. Able-bodied (AB) participants were recruited as controls. This study was approved by the Institutional Review Board at the University of Georgia. We certify that all applicable instructional and governmental regulations concerning the ethical use of human volunteers were followed during the course of this research.

The first part of this study was a cross-sectional study looking at skeletal muscle mitochondrial capacity in MITO participants and comparing them to AB controls. Testing consisted of one session during which oxidative capacity was measured in the forearm flexor muscles of the dominant arm. The NIRS methods and analysis for the recovery kinetics tests used have been previously published with a few adjustments. The second part of this study involved one participant with a MITO performing 18 exercise sessions in one month. This participant was tested for muscle oxidative capacity, muscle endurance, and muscle strength before and after the training sessions were completed.

### NIRS Measurements

The participants were positioned supine on a padded testing table for the duration of the test session. The arm tested was positioned straight out, approximately 45 degrees away from the body. The NIRS probe (Oxymon MK III or Portamon, Artinis Medical Systems, Eisteinweg, The Netherlands) was placed over the surface of the forearm flexor muscles and secured on the forearm with bioadhesive tape and two Velcro straps. Subcutaneous adipose tissue thickness (ATT) was measured at the beginning of each testing session utilizing B-Mode imaging (LOGIQ e; GE Healthcare, Atlanta, GA, USA). The NIRS probe optical distances (either 2, 3, or 4 cm) were adjusted for each participant based on their ATT in order to obtain optimal penetration depth into the skeletal muscle tissue and a signal-to-noise ratio. The difference in signal from the 760 and 850 nm light detectors was used as the indicator of tissue oxygenation. A Hokanson vascular cuff (SC 5 or SC 7 depending on arm circumference) was wrapped around the distal end of the upper arm, proximal to the elbow and the NIRS optode. The cuff was fit to the circumference of the limb. The vascular cuff was attached to a Hokanson AG101 Rapid Cuff Inflation system (Hokanson E20 control box and AG101 compressor). Participants were asked to refrain from moving in order to avoid motion artifact during testing.

Prior to performing exercise, three resting arterial occlusions (30 s on/30 s off) were taken. The average slope of these cuffs provided a measure of resting oxygen consumption (mVO_2_). The slopes were normalized to the physiological calibration (as described below).

Voluntary exercise was used to increase metabolic rate. A dumbbell or weight pouch was used for MITO and control participants to complete forearm curls. The participant’s arm remained flat on the table, and the weight was handed to them when it was time for them to complete the exercise. With the forearm lying supine on the table, the participants curled their wrists towards the forearm continuously at a consistent rate. When the exercise ended, the weight was removed from their hand.

Tissue oxygenation values were normalized using physiological calibration. The participant performed 15 s of voluntary exercise followed by 3–5 min of arterial cuff occlusion. The exercise acted to increase metabolic rate within the muscle, and the cuff was applied until the oxygenated myoglobin/hemoglobin signal reached a steady state (for at least 1 min) value, which represented the relative 0% oxygenation level. Upon the release of the cuff, the peak value from the resultant hyperoxygenation was used to represent the relative 100% oxygenation level of the tissue.

mVO_2_max was measured as previously described [18]. Participants performed 5–25 s of voluntary exercise. The exercise lasted between 5 and 25 s, depending on how quickly the participant de-saturated to 30% of their physiological range, which was determined by physiological calibration. The weight used ranged from 1 to 3 kg and was determined individually for each participant based on his or her ability to produce continuous vigorous muscle contractions. NIRS-measured recovery rates have been shown to be independent of the amount of muscle activation. Exercise was immediately followed by a series of short duration cuffs as follows: cuffs 1 to 5 (5 s on/6 s off), cuffs 6 to 10 (7 s on/7 s off), cuffs 11 to 15 (10 s on/10 s off), and cuffs 16–20 (10 s on/20 s off) over 5 min. A metabolic rate (mVO_2_) was determined for each ischemic cuff. mVO_2_max was calculated as the exponential rate constant of the mVO_2_ measurements during the recovery from exercise. mVO_2_max measurements were performed three times and averaged.

Oxygen desaturation during exercise was measured as the difference between resting oxygen saturation and the oxygen saturation at the end of the exercise. This value was then expressed as a percentage of the participant’s physiological calibration. The slope of the first post exercise cuff occlusion represented the initial post exercise oxygen consumption rate (post exercise mVO_2_). This slope indicates the fastest rate of oxygen consumption occurring as a result of the voluntary exercise performed.

Specific muscle endurance

For the participant who performed endurance training, muscle-specific endurance was determined as measured previously [28]. While positioned supine, the participant laid with the forearm approximately 60° from the body and strapped down. Two electrodes were placed on the brachioradialis muscle, with an accelerometer positioned in between them on the thickest part of the forearm. The NMES was used to stimulate muscle contractions at 2 Hz, 4 Hz, and 6 Hz sequentially for 3 min each at 30 mA. A triaxial accelerometer (WAX3, Axivity Ltd., Newcastle upon Tyne, United Kingdom) was used to measure the magnitude of the first vibration of each contraction. The endurance index for each frequency was the percentage of the acceleration at the end of each session divided by the highest vibration during 2 Hz. Strength was also measured with a handgrip ergometer.

Muscle-specific endurance training

Participant #7 performed endurance training consisting of 18 sessions over 34 days. All training and testing were performed on the non-dominant forearm. The training program was based on similar studies [29]. Wrist curls were performed with handheld dumbbells ranging from 1.36 to 2.27 kg in weight. The participant was instructed on how to perform wrist curls with a full range of motion and with their forearm. Each training session was performed at home but with email feedback on the same day as the session. The participant was instructed to train for 30 min but to take breaks to recover if needed. Changes in weight and contraction frequency were decided by the study team with the participant, based on symptom feedback.

Analysis

Data are presented as means and standard deviations. Comparisons between groups were made using independent *t* tests. Significance was assumed with a *p* value < 0.05.

## 5. Conclusions

In conclusion, in vivo assessment of skeletal muscle mitochondrial capacity (mVO_2_max) in patients with mitochondrial disease showed no differences in comparison to controls. The NIRS-based measurements used in this study were noninvasive and provided a comprehensive evaluation of muscle oxidative metabolism suitable for clinical populations. This study extended the NIRS methods to study people with mitochondrial myopathies. While this study showed that the muscles in the MITO patients could consume oxygen similarly to the able-bodied controls, these results are in contrast to some previous studies. Potential differences could be due to the choice of arm muscles in this study versus leg muscles in previous studies. Arm muscles have lower mVO_2_max values compared to leg muscles, perhaps related to reduced activity levels in the arms relative to the legs in most people. Lower mVO_2_max values could better allow for mitochondrial compensation. Mitochondrial compensation through increased mitochondrial mass has been shown in murine models of mitochondrial myopathies. In a case study, forearm muscle endurance training improved muscle function in a person with a MITO, supporting mitochondrial plasticity in this population. The limitations of this study include the heterogeneity of the MITO group and a single case used for the training program. This study suggests that people with mitochondrial myopathies may not have abnormal skeletal muscle mitochondrial activity. This study also suggests that people with MITOs can respond to endurance training by increasing their skeletal muscle mitochondrial capacity.

## Figures and Tables

**Table 1:** Characteristics of participants with MITOs and controls.

		Height	Weight	BMI	ATT	IPAQ
		m	km	kg/m^2^	cm	MET-min/wk
MITOs	Mean	1.71	64.9	22.3	0.55	4071
	SD	0.11	12.4	3.8	0.21	4106
Controls	Mean	1.69	62.3	21.9	0.51	3369
	SD	0.08	7.3	2.2	0.13	2022

**Table 2:** Selected quotes from the participant after the training sessions.

Week	Training Session	Training	Comments
1	#4	15 min, 3 min rest, 15 min	“Muscle was starting to fatigue at 13/14 min. Second 15 min my arm felt pretty fatigued from the start. Towards the end of the reps were getting jerky. The ‘recovery’ period is still noticeable 15 min later. Muscle feels tired elbow and wrist a little achy.”
2	#8	15 min, 3–5 min rest, 15 min Weight was 1.82 kg 1 contraction every 5 s	“First 15, it seems later in the day I have more control in reps Not shaky. No pain, 0. I was feeling fatigue about 13 min in. When I stopped for a short break I again feel like my arm is weak. Recovery 30 s although I feel unsteady sensation. Second 15, I am noticing a better rep since I began this study. The range of rep is considerably wider. Just the thought of this as I sat lifting up and down up and down. No pain, 0, Weakish when finished.”
3	#15	30 min no break Weight was 1.82 kg 1 contraction every 4 s	“I did a 30 min session with no break. I started to feel fatigued at about 23 min. Pain level 0. No shakiness or weak feeling.”
4	#18 Final session	30 min no break Weight was 1.82 kg 1 contraction every 4 s	“Very interesting. First of all, I really did not experience fatigue. I felt like I was exercising, but not needing a break. I stopped the session and felt completely recovered immediately.”

**Table 3:** Subject characteristics.

ID	Age	Sex	BMI	ATT	IPAQ	Mitochondrial Diagnosis			
						Syndrome	Abnormal	Biochemical	Mitochondrial
							Biopsy	Abnormalities	DNA Mutation
1	59	F	23.7	0.40	378	MM	Yes; complex I defect	None	None
2	34	F	16.1	0.44	3178	MELAS	Not conducted	None	mtDNA 3243 A>G mutation
3	44	F	22.9	0.68	522	MM	Yes, RRF complex I defect	Elevated CPK	mtDNA deletion in muscle
4	46	F	27.5	0.80	997	MM	Yes; RRF no enzymology	Elevated CPK	mtDNA deletion in muscle
5	24	M	24.2	0.35	8106	Mito Cytopathy	Yes; complex I defect	Elevated lactate and CPK	None to date
6	37	M	18.7	0.36	4291	Leigh Syndrome	Yes; signs of mito proliferation	Elevated lactate and CPK	mtDNA 8993 T>C mutation
7	47	F	23.1	0.80	11025	KSS	Yes; complex I defect	Elevated lactate	mtDNA deletion

MM = mitochondrial myopathy, MELAS = mitochondrial encephalomyopathy, lactic acidosis, and stroke-like episodes, KSS = Kearns–Sayre Syndrome.

## Data Availability

Data from this study are provided in the Supplementary Materials.

## Supplementary Materials

The following supporting information can be downloaded at: https://www.mdpi.com/article/10.3390/muscles3040033/s1, Data file: MITO1 DATA SUMMARY.
